# Supplementary material for: WordCommentsAnalyzer: A windows software tool for qualitative research
Source: F1000Res. 2018 Sep 4;7:536. Originally published 2018 May 3. [Version 2] doi: 10.12688/f1000research.14819.2 (PMC6137411; doi:10.12688/f1000research.14819.2)
Supplement: Supplementary file 3 [file f1000research-7-17614-s0002.tgz › b2a79496-4ada-47c4-9682-a99ce867836d.zip!/Eight Iranian Health Journals/Journal of Knowledge and Health_28.docx]

© Journal of Knowledge and Health. This work is licensed under CC BY 4.0 (https://creativecommons.org/licenses/by/4.0/).

Volume & Issue: دوره 6، شماره 2: 1390

Title: اثربخشی درمان گروهی پذیرش و تعهد بر هراس اجتماعی دانشجویان

http://knh.shmu.ac.ir/index.php/site/article/view/100

مقدمه: درمان پذیرش و تعهد، یکی از درمان‌های رفتاری نسل سوم درزمینه درمان هراس اجتماعی است. این تحقیق به‌منظور بررسی اثربخشی درمان گروهی پذیرش و تعهد بر هراس اجتماعی دانشجویان انجام گرفته است. مواد و روش‌ها: این تحقیق از نوع مطالعات مداخله‌اي است. 24 دانشجوي مبتلا به اختلال هراس اجتماعی، بر‌اساس مصاحبه‌ باليني و پرسش‌نامه‌ هراس و اضطراب اجتماعی از میان دانشجویان مؤسسه آموزش عالی شمال شهرستان آمل (دانشگاه شمال) انتخاب شدند و به‌طور تصادفی در دو گروه مداخله و شاهد قرار گرفتند. درمان گروهی پذیرش و تعهد در 10 جلسه 90 دقيقه‌اي درمورد گروه مداخله انجام شد و گروه شاهد، درماني دريافت نكرد. نمرات پیش‌آزمون، پس‌آزمون و پیگیری یک‌ماهه بعد از دوره درمانی درمورد هر دو گروه، با روش تحليل واريانس چندمتغيري و t  مستقل مورد تحلیل قرار گرفت. نتایج: نتایج نشان داد که در انتهای درمان، نمرات اضطراب اجتماعی گروه مداخله نسبت‌به گروه شاهد به‌طور معناداری کاهش یافته است (05/0>P) و این کاهش در مدت پیگیری یک ماه نیز تغییر معناداری نیافته است (05/0P>). نتیجه‌گیری: درمان گروهی پذیرش و تعهد، بر درمان اختلال اضطراب اجتماعی در میان دانشجویان مؤثر است.

Title: تحول و شکوفایی معنوی، فعالیت‌های اجتماعی مذهبی و کیفیت زندگی در معتادان گمنام

http://knh.shmu.ac.ir/index.php/site/article/view/107

مقدمه: معتادان گمنام، انجمن متشکل از مردان و زنانی است که برای رهایی از اعتیاد، تجاربشان را به اشتراک می‌گذارند و از برنامه‌های معنوی 12 قدمی تبعیت می­کنند. پژوهش حاضر با هدف یافتن سهم ابعاد مؤلفه‌های معنويت در پیش‌بینی کیفیت زندگی اعضای انجمن معتادان گمنام انجام گرفته است. مواد و روش­ها: در این مطالعه پس‌رویدادی، 96 مرد داوطلب از اعضای انجمن معتادان گمنام شهرستان شاهرود، در محدوده سنی 20-40 سال، که حداقل 6 ماه پرهیز از مواد داشته‌اند، وارد مطالعه شدند. ابزار جمع‌آوری داده‌ها، پرسش‌نامه‌های مقیاس کیفیت زندگی سازمان بهداشت جهانی و مقیاس تجربه معنوی بود و براي تحلیل داده‌ها از رگرسیون خطی با استفاده از نرم‌افزار SPSS، استفاده شد. نتایج: شکوفایی و فعالیت معنوی و فعالیت‌های اجتماعی مذهبی، به‌عنوان ریزمؤلفه‌های معنویت، به‌طور معناداری در پیش­بینی کیفیت زندگی اعضای انجمن معتادان گمنام نقش داشتند. ریزمؤلفه شکوفایی و فعالیت معنوی 25/0(25/0=R2، 01/0>P) و ریزمؤلفه فعالیت‌های اجتماعی مذهبی در ترکیب با ریزمؤلفه اول 34/0(34/0=R2،01/0>P) از واریانس کیفیت زندگی را تبيین کردند.نتیجه‌گیری: به‌نظر‌می‌رسد که شرکت داشتن فعال معتادان در انجمن معتادان گمنام و تبعیت از اصول 12گانه، باعث تحول و شکوفایی فعالیت معنوی و فعالیت‌های اجتماعی مذهبی افراد مي‌شود که زمینه ارتقاي کیفیت زندگی افراد را فراهم می‌کند

Title: مقايسه ميزان ماتريکس متالوپروتئيناز-9 در سرم و پلاسماي بيماران مبتلا به سرطان پستان

http://knh.shmu.ac.ir/index.php/site/article/view/109

مقدمه: ماتريکس متالوپروتئيناز-9 (MMP-9) به‌تازگي به‌عنوان يکي از ژن‌هاي مارکر براي بسياري از سرطان‌ها گزارش شده است. باتوجه به يافته‌هاي قبلي درمورد اين ژن، در اين مطالعه به مقايسه ارتباط ميزان پلاسمايي و سرمي اين آنزيم با خصوصيات باليني بيماران مبتلا به سرطان پستان پرداختيم. مواد و روش‌ها: در اين مطالعه مورد- شاهدي، 114 بيمار مبتلا به سرطان پستان به همراه 87 فرد سالم مورد تحقيق قرار گرفتند. براي بررسي ميزان MMP-9 در سرم و پلاسماي بيماران از روش زيموگرافي ژلاتين و کيت بايو رد (Bio-Rad, Richmond, CA) استفاده شد. نتایج: ميزان MMP-9 پلاسمايي در بيماران بسيار بيشتر از افراد سالم بود و تفاوت آن بيشتر از ميزان تفاوت MMP-9 سرمي اين دو گروه بود (001/0P<). ارتباط معناداري بين افزايش ميزان MMP-9 پلاسمايي و متاستاز به غدد لنفاوي (019/0=P، 4/3OR,) و تهاجم وريدي (033/0=P، 14/4OR,) بيماران مورد مطالعه وجود داشت. ارتباط خاصي بين ميزان MMP-9 سرمي و متاستاز لنفاوي يا تهاجم وريدي مشاهده نشد. نتیجه‌گیری: نتايج مطالعه نشان مي‌دهد که براي تشخيص زودهنگام بدخيمي سرطان پستان، ميزان پلاسمايي اين آنزيم، مناسب‌تر از ميزان سرمي آن است.

Title: هورمون رشد، شاخص مقاومت انسولینی، نیمرخ لیپیدی و عملکرد قلبی- تنفسی در مردان جوان چاق و لاغر غیرفعال: ارتباط با سطوح گرلین آسیل‌دار پلاسما

http://knh.shmu.ac.ir/index.php/site/article/view/111

مقدمه: گرلین پلاسما تحت تأثیر وضعیت تغذیه‌ای فرد مي‌باشد و تصور می‌شود که در تنظیم کوتاه‌مدت و دراز‌مدت خوردن غذا و وزن بدن ایفای نقش مي‌كند. مطالعه حاضر با هدف مقایسه هورمون رشد، شاخص مقاومت انسولینی، نیمرخ لیپیدی و عملکرد قلبی- تنفسی در مردان چاق ‌و لاغر غیرفعال و تعیین ارتباطات آن‌ها با سطوح گرلین آسیل‌دار پلاسما اجرا شد. مواد و روش­ها: در اين مطالعه مقايسه‌اي، 19 مرد جوان چاق (نمایه توده بدنی kg/m2 0/31) و 19 مرد جوان لاغر (نمایه توده بدنی kg/m25/18) که طی 6 ماه گذشته هیچ‌گونه فعالیت بدنی منظمی نداشتند، به‌عنوان آزمودنی انتخاب شدند. پس از 12 ساعت ناشتایی (در ساعت 8 صبح)، نمونه‌های خون براي تعیین سطوح گرلین آسیل‌دار، انسولین، هورمون رشد، گلوکز و نیمرخ لیپیدی جمع‌آوری شد. حداکثر اکسیژن مصرفی (به‌عنوان شاخص عملکرد قلبی- تنفسی) آزمودنی‌ها نیز سنجيده شد. نتایج: آزمودنی‌های چاق نسبت‌به لاغر، از سطوح انسولین و HOMA-IR (شاخص مقاومت انسولینی) بالاتری برخوردار بودند، درحالی‌که سطوح گرلین آسیل‌دار، هورمون رشد و حداکثر اکسیژن مصرفی در آنان پایین‌تر بود (01/0‌>‌P). هیچ‌گونه تفاوت معناداری بین مقادیر فشارخون سیستولیک و دیاستولیک، گلوکز ناشتا و نیمرخ لیپیدی در دو گروه مشاهده نشد (01/0<P). غلظت گرلین آسیل‌دار، رابطه معکوسي با وزن کل، درصد چربی بدن، نمایه توده بدنی، انسولین و شاخص مقاومت انسولینی و رابطه مستقیمي با سطوح هورمون رشد و حداکثر اکسیژن مصرفی داشت (01/0‌>‌P). رابطه معناداری بین غلظت گرلین آسیل‌دار و مقادیر فشارخون سیستولیک و دیاستولیک، گلوکز ناشتا و نیمرخ لیپیدی مشاهده نشد (01/0‌<‌P).نتیجه‌گیری: مردان جوان چاق و لاغر غیرفعال از سطوح گرلین آسیل‌دار، هورمون رشد، انسولین، شاخص مقاومت انسولینی، عملکرد قلبی- تنفسی و درصد چربی بدنی متمایزی برخوردار بودند و به‌نظر‌می‌‌رسد درصد چربی بدن، انسولین و هورمون رشد، قوی‌ترین عوامل تعیین‌کننده سطوح گرلین آسیل‌دار هستند

Title: تعيين هويت انگل بيماري ليشمانيوز جلدي (سالك) به روش PCR در مناطق آلوده استان گلستان

http://knh.shmu.ac.ir/index.php/site/article/view/112

مقدمه: تاکنون تقسيم‌بندي و تعيين گونه انگل لیشمانیازیس به‌طورغالب براساس مشخصات باليني، پراكندگي جغرافيايي و گاهي اندازه انگل بوده است. باتوجه به محدودیت‌های این تقسیم‌بندی اين پژوهش جهت تعيين وضعيت اپيدميولوژيك و تعيين دقیق گونه‌های انگل براساس روش­هاي مولكولي و تعيين گونه غالب انگل در مناطق آلوده استان گلستان به‌عنوان يكي از كانون­هاي ليشمانيوز جلدي نوع مرطوب (روستايي) در ايران طراحي شده است.  مواد و روش­ها: اين پژوهش یک مطالعه توصيفي- تحليلي می‌باشد. جامعه مورد مطالعه آن را 63 بيمار داراي زخم­هاي مشكوك به سالك تشكيل مي‌دهند كه در سال‌هاي 83-86 به آزمايشگاه‌ها و خانه‌هاي بهداشت روستايي مناطق آلوده استان گلستان مراجعه كرده بودند. ابزار جمع‌آوری داده‌ها، پرسش‌نامه‌اي حاوي متغیرهای دموگرافيك مانند: سن، جنس و محل سكونت و نيز متغیرهای باليني مانند: شكل، نوع، محل، تعداد و اندازه زخم، سابقه ابتلاي قبلي وجود اسكار، تعداد و محل اسكار بود. همچنین هم‌زمان با تکمیل پرسش‌نامه از نمونه زخم بيماران لام مستقيم تهيه شد و نيز نمونه‌اي جهت كشت در محيط كشت دي فازيك N.N.N (نویی، مک نیل، نیکول) تهيه گرديد. پس از رشد انگل در محيط كشت، DNA انگل استخراج و سپس آزمايش‌هاي PCR انجام شد. نتايج: رابطه آماری معناداری بین گونه لیشمانیا و قومیت و جنس وجود داشت (05/0P<)، به­طوری­که 100% )54 نفر) موارد ابتلا در قومیت ترکمن را لیشمانیا ماژور و 100% (3 نفر) موارد مبتلا در قومیت سیستانی را لیشمانیا تروپیکا تشکیل می‌دادند. در قوم فارس 3/33% (2 نفر)گونه ماژور و 7/66% (4 نفر)گونه تروپيكا ديده شد. اكثر بيماران، متعلق به مناطق روستايي شهرستان گنبد بودند. اين بيماري در هر دو جنس ديده شد، ولي تعداد مردان مبتلا (1/57%) بيش از زنان (9/42%) گزارش شده است. نتيجه­گيري: اين پژوهش نشان داد كه گونه غالب انگل در مناطق آلوده استان گلستان ليشمانيا ماژور و بيماري ليشمانيوز جلدي آن از نوع روستايي يا مرطوب مي‌باشد

Title: کاهش رفتارهاي دردي آزمون فرمالين با تزریق مرفين به داخل هسته پاراژیگانتوسلولاریس موش صحرايي

http://knh.shmu.ac.ir/index.php/site/article/view/114

مقدمه: تزریق سیستمیک مرفین سبب بي‌دردي در آزمون فرمالین می‌شود، ولي هسته‌ها و مراکز مغزي که اين بي‌دردي را وساطت مي‌کنند، مشخص نشده است؛ بنابراين در اين مطالعه اثرات تزريق داخل هسته پاراژیگانتوسلولاریس جانبی (LPGI) مرفین، روي پاسخ رفتاري به درد در آزمون فرمالين بررسي شده است. مواد و روش‌ها: در اين مطالعه‌ي رفتاري از مدل درد التهابي به‌وسيله فرمالين در موش‏هاي صحرايي سفيد، در محدوده وزني 200- 300 گرم استفاده شد. با عمل جراحي استروتاکسی، كانول راهنما در داخل هستهLPGI  قرار گرفت و پس از يك هفته استراحت، آزمون فرمالین با تزريق 50 ميكروليتر محلول فرمالين 2% به زير پوست پنجه پاي حيوان انجام شد. نتایج: تزريق مرفین به داخل LPGI سبب کاهش شدید رفتارهاي دردي در مرحله فاز 1، اينترفاز و فاز 2 آزمون فرمالين شد (دوز 50 نانومول). در مقايسه با تزريق داخل LPGI مرفين که رفتارهاي دردي را در فاز 1، اينترفاز و فاز 2 آزمون فرمالين شديداً کاهش داد (001/0>P)، تزريق جانبي‌تر نسبت‌به LPGI مرفین، هيچ تأثيري روي رفتارهاي دردي آزمون فرمالين نداشت. نتیجه‌گیری: تزريق داخل LPGI مرفین سبب کاهش شدید رفتارهاي دردي در مرحله فاز 1، اينترفاز و فاز 2 آزمون فرمالين شد که پیشنهاد می‌کند گیرنده‌های اپیویئدی در LPGI سبب مداخله با مکانيسم‌هاي رفتارهاي دردي در آزمون فرمالين به‌عنوان درد التهابی مي‌شود

Title: شیوع سوءِ‌تغذیه در دانش‌آموزان مناطق محروم استان خراسان جنوبی

http://knh.shmu.ac.ir/index.php/site/article/view/115

مقدمه: سوءِتغذيه در کل جهان به‌خصوص در کشورهای در حال توسعه از مهم‌ترین مشکلات سلامت عمومی به‌شمارمی‌رود که اثرات نامطلوبی بر رشد و تکامل ذهنی کودکان ایجاد می‌كند. مطالعات متعدد در ایران حاکی از وجود سوءِتغذيه در نقاط مختلف است، اما به‌ندرت مطالعه‌اي به‌منظور بررسي وضعيت تغذيه‌اي كودكان در مناطق محروم انجام شده است. مواد و روش­ها: این مطالعه مقطعی بر روی 1211 دانش‌آموز 7- 14 ساله (مدارس ابتدایی و راهنمایی‌) از مناطق محروم استان خراسان جنوبی انجام شد که به روش نمونه‌گیری خوشه‌ای چندمرحله‌ای سهمی از مناطق شهری و روستایی 5 شهرستان تحت پوشش استان انتخاب شده بودند. اندازه‌گیری قد و وزن را کاردان و کارشناسان طرح انجام دادند و برای بررسی سوءِتغذيه از 3 شاخص وزن به سن (کم‌وزنی)، وزن به قد (لاغری) و قد به سن (کوتاه‌قدی) استفاده شد. داده‌ها با استفاده از نرم‌افزار SPSS و EP Info 2000 و آزمون کای اسکوئر در سطح معناداری 05/0α= مورد تجزیه و تحلیل قرار گرفتند. نتایج: از 1211 دانش­آموز 2/54% پسر و بقیه دختر بودند. 6/70% دانش‌آموزان کم‌وزن، 70% کوتاه‌قد و 20% لاغر بودند. بیشترین موارد کم‌وزنی، کوتاه‌قدی و لاغری از نوع خفیف و کمترین موارد از نوع شدید بود. نتايج نشان داد كه شيوع كم‌وزني و كوتاهي قد با افزايش سطح تحصيلات مادران به‌طور معناداري كاهش مي‌يابد (001/0P<). نتیجه‌گیری: درصد بالایی از دانش‌آموزان مناطق محروم از سوءِتغذيه رنج می‌برند و اقداماتی مانند افزایش سواد مادران و توجه بیشتر به بهبود وضعیت تغذیه‌ای دانش‌آموزان در خانواده‌های محروم توصیه می‌شود.

Title: بررسي کمیت و کیفیت پسماند مطب‌هاي دندانپزشكي

http://knh.shmu.ac.ir/index.php/site/article/view/120

مقدمه: امروزه يكي از مهم‌ترين معضلات زيست‌محيطي، مواد زايد توليدي در مراکز دندانپزشکي است كه به‌علت داشتن عوامل خطرناك، سمي و بيماري‌زا از اهميت خاصي برخوردار است. هدف اين مطالعه بررسي و آناليز مواد توليدي در مراکز دندانپزشکي شهر شاهرود مي‌باشد. مواد و روش‌ها: در اين مطالعه توصيفي، کل مطب‌های دندانپزشکی شهر شاهرود انتخاب شدند و از هر مطب، سه نمونه در انتهاي 3 روز كاري متوالي (‌روزهاي دوشنبه، سه‌شنبه و چهارشنبه هر هفته)، 3 نمونه برداشت شد. نمونه‌ها به‌صورت دستي، جداسازي و به 64 جزءِ مختلف تفكيك و با استفاده از ترازوي آزمايشگاهي وزن شدند. در مرحله بعد، اجزاي وزن‌شده بر‌اساس ويژگي و پتانسيل خطرزايي دسته‌بندي شدند. نتایج: ميزان كل پسماند توليدي در مطب‌هاي دندانپزشكي شهر شاهرود برابر با 48/2425 كيلوگرم در سال مي‌باشد. سهم توليد پسماندهاي عفوني، پسماندهاي شبه‌خانگي، پسماندهاي شيميايي و دارويي و پسماندهاي سمي به‌ترتيب برابر با 0/46%، 8/43%، 2/9% و 0/1% مي‌باشد.نتیجه‌گیری: دررابطه‌با مديريت بهينه‌اي دندانپزشكي پيشنهاد بر اين است كه علاوه بر آموزش دندانپزشكان جهت انجام فعاليت‌هاي مربوط‌به كاهش، جداسازي و يا بازيافت پسماند در داخل مطب؛ جمع‌آوري، حمل‌و‌نقل و دفع هركدام از بخش‌هاي پسماندهاي دندانپزشكي به‌صورت جداگانه و مطابق با دستورالعمل‌ها و ضوابط مربوطه انجام گيرد
